# Optogenetic Stimulation Shifts the Excitability of Cerebral Cortex from Type I to Type II: Oscillation Onset and Wave Propagation

**DOI:** 10.1371/journal.pcbi.1005349

**Published:** 2017-01-24

**Authors:** Stewart Heitmann, Michael Rule, Wilson Truccolo, Bard Ermentrout

**Affiliations:** 1 Department of Mathematics, University of Pittsburgh, Pittsburgh, Pennsylvania, USA; 2 Department of Neuroscience, Brown University, Providence, Rhode Island, USA; 3 U.S. Department of Veterans Affairs, Center for Neurorestoration and Neurotechnology, Providence, Rhode Island, USA; UCL, UNITED KINGDOM

## Abstract

Constant optogenetic stimulation targeting both pyramidal cells and inhibitory interneurons has recently been shown to elicit propagating waves of gamma-band (40–80 Hz) oscillations in the local field potential of non-human primate motor cortex. The oscillations emerge with non-zero frequency and small amplitude—the hallmark of a type II excitable medium—yet they also propagate far beyond the stimulation site in the manner of a type I excitable medium. How can neural tissue exhibit both type I and type II excitability? We investigated the apparent contradiction by modeling the cortex as a Wilson-Cowan neural field in which optogenetic stimulation was represented by an external current source. In the absence of any external current, the model operated as a type I excitable medium that supported propagating waves of gamma oscillations similar to those observed in vivo. Applying an external current to the population of inhibitory neurons transformed the model into a type II excitable medium. The findings suggest that cortical tissue normally operates as a type I excitable medium but it is locally transformed into a type II medium by optogenetic stimulation which predominantly targets inhibitory neurons. The proposed mechanism accounts for the graded emergence of gamma oscillations at the stimulation site while retaining propagating waves of gamma oscillations in the non-stimulated tissue. It also predicts that gamma waves can be emitted on every second cycle of a 100 Hz oscillation. That prediction was subsequently confirmed by re-analysis of the neurophysiological data. The model thus offers a theoretical account of how optogenetic stimulation alters the excitability of cortical neural fields.

## Author Summary

Optogenetic stimulation is increasingly used as a surrogate for endogenous activity to probe neural dynamics. Our model shows that optogenetic stimulation which predominantly recruits inhibitory neurons can dramatically alter the neural dynamics from type I excitability (integrators) to type II excitability (resonators). We claim that this phenomenon explains the seemingly paradoxical co-existence of propagating waves (a hallmark of type I excitability) and the onset of oscillations with small amplitude (a hallmark of type II excitability) observed in macaque motor cortex. The model provides a theoretical account of how optogenetic stimulation alters the excitability of neural tissue. As such, it predicts that propagating gamma waves can also emerge from 100 Hz oscillations at the site of the optogenetic stimulation. This prediction was confirmed by a subsequent analysis of previously published neurophysiological data.

## Introduction

**Fig 1:**
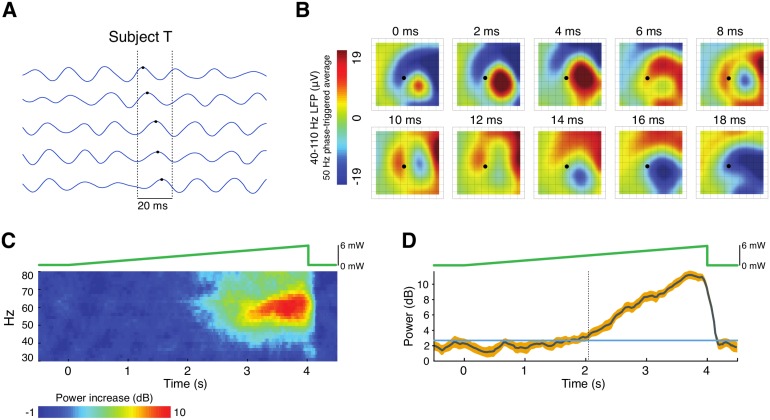
Optogenetically induced gamma band (40–60 Hz) oscillations in primate motor cortex, redrawn from [1]. *A:* Gamma oscillations in the local field potentials at five recording sites on the microelectrode array for subject T. The oscillation phase has a spatial gradient that indicates wave propagation. Black dots indicate the peaks of one gamma cycle across neighboring electrodes; *B:* Optogenetic stimulation induces expanding waves, as summarized in the phase-triggered average of gamma (40–110 Hz) spatial field potential, based on the phase of the optogenetically-induced 50 Hz gamma oscillation. The dot indicates the point where the fiber optic light source was surgically inserted. The tip of the optical fiber was likely slanted to the right of this point, corresponding to the origin of the waves. *C:* Trial-averaged spectrogram of the local field potential when the optical stimulation was ramped from 0 mW to 6 mW over 4 seconds. The mean power within each frequency band for the 500 ms preceding stimulation was subtracted (in dB) from the power during stimulation to enhance visualization of the optogentically-induced changes. *D:* Power of the oscillations in the local field potential during the ramp protocol.

Lu and colleagues [1] recently transduced small regions of primary motor (M1) and ventral premotor (PMv) cortices of macaque monkeys using red-shifted opsin C1V1(T/T). They found that constant optical stimulation of the targeted tissue induced intrinsic gamma-band (40–80 Hz) oscillations in the local field potential (Fig 1A). The gamma oscillations were manifest in 4x4 mm^2^ microelectrode recordings as patterns of concentric rings and spiral waves that propagated into the surrounding tissue well beyond the stimulation site (Fig 1B). When the optogenetic stimulation was slowly ramped from zero, the oscillations emerged abruptly at a non-zero frequency (Fig 1C) with low amplitude (Fig 1D). Lu et al recognized that these two characteristics are consistent with a dynamical system undergoing a supercritical Hopf bifurcation [1].

The supercritical Hopf bifurcation is the hallmark of type II neural excitability [2]. It encapsulates the dynamical properties of neurons that can fire arbitrarily small spikes but have a relatively fixed firing rate [3]. Type I neurons, on the other hand, are characterized by fixed amplitude spikes. Those dynamics can arise from a subcritical Hopf bifurcation or a saddle-node bifurcation on an invariant circle (SNIC) [2, 4]. The SNIC bifurcation allows arbitrarily small firing rates whereas the subcritical Hopf bifurcation has relatively fixed firing rates. Izhikevich [4] characterizes type I neurons as *integrators* and type II neurons as *resonators*. In the present study, we apply the classifications of type I and II excitability in single neurons to the excitability of populations of neurons in spatially extended neural media. Those same classifications determine how well an excitable medium can sustain propagating waves of activity. For the case of a type I medium, small disturbances to the resting state produce large amplitude responses that readily propagate over long distances. Whereas small disturbances in type II media typically elicit only small responses that tend not to propagate. There is an exception however—type II media can become highly excitable when the time course of the recovery variable is substantially slower than that of excitation [5]. In such cases, the dynamics are that of a relaxation oscillator [6] which is capable of supporting propagating waves because of its explosive response to small inputs.

Lu et al’s [1] observation that optogenetically-induced gamma waves propagate far beyond the site of stimulation suggests that the cortical tissue normally operates as a type I excitable medium. However this seems to contradict the finding that the optogenetically stimulated tissue operates as a type II excitable medium as revealed by the ramped stimulus protocol. This apparent contradiction offers a glimpse into the effect of optogenetic stimulation on the excitability of neural tissue. We explored the theoretical implications by simulating the propagation of gamma waves in a continuum neural field model of cortex. The model comprised of recurrently connected populations of excitatory and inhibitory neurons which were driven by an external current source that represented the ionic currents induced by optogenetic stimulation. We sought to determine (i) the conditions under which a type II excitable medium could sustain propagating waves of gamma oscillations and (ii) whether the act of optogenetic stimulation could transform a type I excitable medium into type II that produces graded oscillations.

## Models

The cortex was modeled as a continuum neural field of recurrently connected populations of excitatory and inhibitory neurons following the methods of Wilson and Cowan [7, 8]. The neural field represents the spatiotemporal intensity function for neural firing at spatial position *x* firing a spike at time *t*. The excitatory and inhibitory populations were treated as separate but interconnected neural fields. The equations governing their firing rates were defined as

$$\tau_{e}\;\dot{U_{e}}=-U_{e}+F\left(K_{ee}*U_{e}-K_{ei}*U_{i}+J_{e}-b_{e}\right)$$ (1)

$$\tau_{i}\;\dot{U_{i}}=-U_{i}+F\left(K_{ie}*U_{e}-K_{ii}*U_{i}+J_{i}-b_{i}\right)$$ (2)

where *U*_*e*_(*x*, *t*) and *U*_*i*_(*x*, *t*) represent the mean firing rates of excitatory and inhibitory populations respectively. The local field potential (LFP) was defined as a weighted sum of the local mean firing rates,

$$\text{LFP}\left(x,t\right)=0.8\;U_{e}\left(x,t\right)+0.2\;U_{i}\left(x,t\right),$$ (3)

with the contribution of excitatory cells weighted four times that of inhibitory cells. This weighting reflects the higher prevalence of excitatory cells as well as their greater contribution to the electric field due to the arrangement of their dipoles. The firing rates were related to synaptic input by the sigmoidal function,

F(u)=1/(1+exp(-u)), (4)

where *u*(*x*, *t*) represents the local synaptic input at spatial position *x* at time *t*. The local synaptic input was computed as a weighted sum of excitatory and inhibitory spiking activity in the immediate vicinity plus external currents *J*_*e*_(*x*, *t*) and *J*_*i*_(*x*, *t*) that represented the additional synaptic currents induced by optogenetic stimulation. Spatial summation is denoted by the convolution operator,

K(x)*U(x,t)=∫K(x-y)U(x,t)dy, (5)

where *K*(*x*) is the spatial density profile of the lateral neural projections. That spatial profile was assumed to be Gaussian with distance,

$$K_{ei}\left(x\right)=\frac{k_{ei}}{\sigma \sqrt{\pi }}\exp\left(\frac{-x^{2}}{\sigma^{2}}\right),$$ (6)

where *σ* is the spatial spread and *k*_*ei*_ is the weight associated with the specific connection type indicated by the subscript (inhibitory-to-excitatory in this case). The connection weights *k*_*ee*_, *k*_*ei*_, *k*_*ie*_, *k*_*ii*_ differed by connection type and connections emanating from excitatory populations were assumed to have twice the spatial spread of those emanating from inhibitory populations. See Table 1 for specific parameter values. Parameters *b*_*e*_ and *b*_*i*_ represent the firing thresholds of the excitatory and inhibitory neurons. Parameters *τ*_*e*_ and *τ*_*i*_ are the time constants of excitation and inhibition.

**Table 1:** Default parameters of the neural field model.

Parameter	Description
*k*_*ee*_ = 15	excitatory-to-excitatory weight
*k*_*ei*_ = 15	inhibitory-to-excitatory weight
*k*_*ie*_ = 15	excitatory-to-inhibitory weight
*k*_*ii*_ = 7	inhibitory-to-inhibitory weight
*σ*_*e*_ = 0.2	spread of excitation (mm)
*σ*_*i*_ = 0.1	spread of inhibition (mm)
*b*_*e*_ = 4	threshold of excitation
*b*_*i*_ = 4	threshold of inhibition
*τ*_*e*_ = 2	time constant of excitation (ms)
*τ*_*i*_ = 4	time constant of inhibition (ms)
*dx* = 0.01	spatial discretization (mm)

The connections weights and the relative time course of excitation and inhibition both impact the excitability of the neural dynamics which, in turn, effects the capacity of the neural field to support propagating waves. We identified the parameters under which steady optogenetic stimulation replicated the emergence of gamma oscillations in the local field potential. We then explored how far those oscillations propagated away from the stimulation site. See [9] and [10] for reviews of the Wilson-Cowan model. See [11] for a review of continuum neural fields in general.

## Results

**Fig 2:**
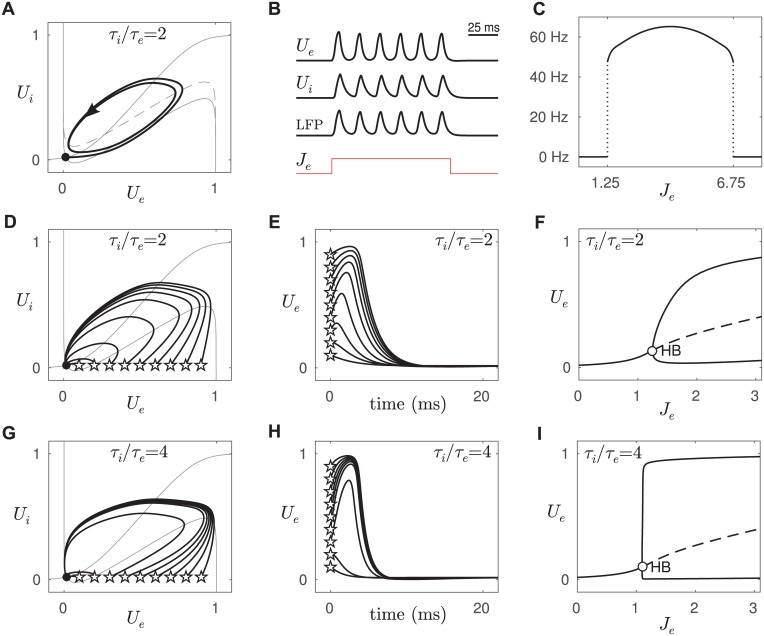
Type II excitability in an isolated pair of excitatory-inhibitory populations. *A:* Phase portrait of the model with *τ*_*i*_ = 4 ms and *τ*_*e*_ = 2 ms. The system is initially at rest (black dot; *U*_*e*_ = 0.017, *U*_*i*_ = 0.020) whereupon a steady injection current (*J*_*e*_ = 2) induces a stable limit cycle (heavy black line) via a supercritical Hopf bifurcation. Nullclines are shown in light gray (excitatory is “cubic”-shaped and inhibitory is “S-shaped”). The dashed nullcline is that of *U*_*e*_ when the injection current is applied. *B:* Corresponding time plots of *U*_*e*_, *U*_*i*_, LFP and the injection current *J*_*e*_. *C:* Frequency of the oscillation as a function of injection current *J*_*e*_. The frequency is always wi1thin the 40–80 Hz gamma band. *D:* Return trajectories for a range of perturbations applied to the resting state. Stars mark the initial conditions. Only the largest perturbations induced large excursions in phase space. *E:* Time plots of the same return trajectories. *F:* Bifurcation diagram showing the envelope of the oscillations in *U*_*e*_ as a function of the injection current. The critical point of the Hopf bifurcation is labelled HB. The dashed line indicates unstable fixed points. *G-I:* Same as panels D-F except that the time constant of excitation has been halved (*τ*_*e*_ = 1 ms). This regime is said to be more excitable because small perturbations produce large responses.

We began by analyzing the excitability of an isolated pair of excitatory-inhibitory populations. The spatial coupling kernels *K*(*x*) in Eqs (1 and 2) were replaced with scalar connection weights (*k*_*ee*_ = 15, *k*_*ei*_ = 15, *k*_*ie*_ = 15, *k*_*ii*_ = 7). The connection weights and threshold parameters (*b*_*e*_ = 4, *b*_*i*_ = 4) were chosen so that the sigmoidal nullcline of the inhibitory population *U*_*i*_ intersected the cubic-shaped nullcline of the excitatory population *U*_*e*_ near the left knee of the cubic (Fig 2A). This particular configuration is known to undergo a supercritical Hopf bifurcation when an external current is applied to the excitatory population [12, 13].

As anticipated, the model produced intrinsic oscillations in the simulated LFP when a steady external current (*J*_*e*_ = 2) was applied to the excitatory cell (Fig 2B). The time constants of excitation (*τ*_*e*_ = 2 ms) and inhibition (*τ*_*i*_ = 4 ms) were chosen so that the frequency of this oscillation always fell within the 40–80 Hz gamma band (Fig 2C). The gamma oscillations emerged at zero amplitude and grew monotonically as the external current was increased (Fig 2F). In all, the isolated pair of excitatory-inhibitory populations replicated the characteristics of gamma oscillations observed by [1] during ramped optogenetic stimulation. The next step was to investigate whether those gamma oscillations would propagate in a spatially extended medium.

The present type II model is only weakly excitable because small perturbations do not induce large responses in *U*_*e*_ (Fig 2D and 2E). Nonetheless, excitability can be enhanced by increasing the time course of inhibition relative to excitation [5]. The same model with *τ*_*e*_ = 1 ms instead of *τ*_*e*_ = 2 ms readily evokes large responses to small perturbations (Fig 2G and 2H). The degree of excitability is evident in the bifurcation structure of *U*_*e*_ versus *J*_*e*_. The amplitude of the oscillation rises gradually in the case of the weakly excitable system (Fig 2F) and explosively in the case of the highly excitable system (Fig 2I). In the next section, we investigate how the degree of excitability of a type II spatial medium governs its ability to sustain propagating waves of gamma oscillations.

### Wave propagation in a type II spatial medium

We modeled a 4 mm long strip of cortex as a type II neural medium using a chain of reciprocally coupled excitatory-inhibitory populations. It represented neural tissue spanning the width of the microelectrode array used by Lu et al [1]. The populations were evenly spaced at intervals of *dx* = 0.01 mm and coupled with a Gaussian spatial density profile Eqs (5 and 6). The Gaussian spread parameters were *σ*_*e*_ = 0.2 mm for excitatory cells and *σ*_*i*_ = 0.1 mm for inhibitory cells. The axons of the excitatory cells thus reached further than those of the inhibitory cells. Optogenetic stimulation was approximated by a focal current source with a square spatial profile (0.4 mm wide) that was centered on the midpoint of the chain (*x* = 0). We surveyed the distance that the waves propagated from the stimulation site for a range of excitatory time constants *τ*_*e*_ while fixing *τ*_*i*_ = 4 ms to preserve the frequency of the gamma oscillations as much as possible. The amplitude of the external current was also fixed (*J*_*e*_ = 1.1). The propagation distance was designated as that point *x* where the maximal value of *U*_*e*_(*x*, *t*) fell below 0.05. Absorbing boundary conditions were imposed at both ends of the chain to prevent waves from reflecting back into the medium.

**Fig 3:**
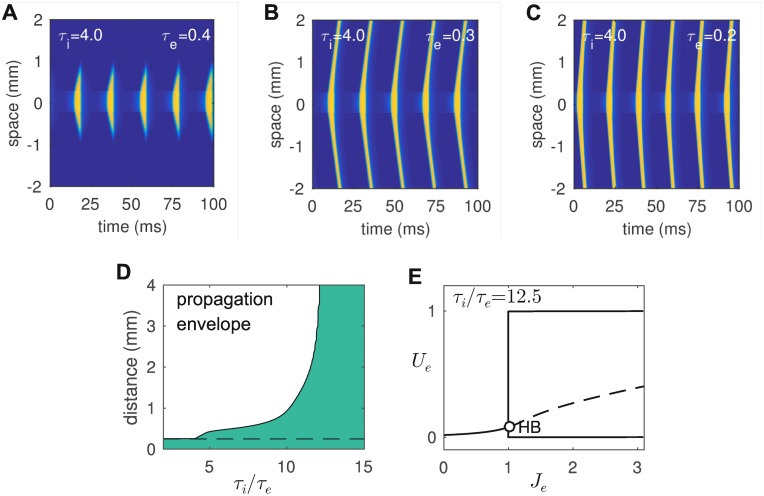
Propagation of LFP waves in one spatial dimension for the model with type II excitability. *A-C:* Space-time plots for media with a range of excitatory time constants, *τ*_*e*_ = {0.4, 0.3, 0.2}. In all cases, steady stimulation (*J*_*e*_ = 1.3) was applied focally to the excitatory cells near the origin (−0.25<*x*<0.25) and the time constant of inhibition was fixed at *τ*_*i*_ = 4 ms. Absorbing boundary conditions were imposed at |*x*| = 3 mm (not shown). *D:* The propagation envelope (shaded region) as a function of the relative time course of inhibition and excitation (*τ*_*i*_/*τ*_*e*_). The dashed line indicates the boundary of the stimulated region. No waves emitted from the stimulated region for *τ*_*i*_/*τ*_*e*_<4. Waves propagated a finite distance for 4 < *τ*_*i*_/*τ*_*e*_ ≲ 12.5. Indefinite wave propagation occurred for *τ*_*i*_/*τ*_*e*_ ≳ 12.5. *E:* Bifurcation diagram for the isolated excitatory-inhibitory model with *τ*_*i*_/*τ*_*e*_ = 12.5. The near-instantaneous rise in the oscillation amplitude at the Hopf bifurcation is markedly different from the slow rise observed in the neurophysiological data.

We found that gamma oscillations failed to propagate at all for *τ*_*i*_/*τ*_*e*_ < 4. Waves that propagated a finite distance (Fig 3A) were observed for values of *τ*_*i*_/*τ*_*e*_ between 4 and approximately 12.5. The propagation distance grew rapidly as *τ*_*i*_/*τ*_*e*_ approached 12.5 and the waves appeared to propagate indefinitely (Fig 3B and 3C) for *τ*_*i*_/*τ*_*e*_ ≳ 12.5 (Fig 3D). The bifurcation diagram (Fig 3E) reveals how the oscillation amplitude rises almost instantaneously for the case of *τ*_*i*_/*τ*_*e*_ = 12.5. Such explosive growth is contrary to the slow rise in gamma power that is observed in the optogenetic ramp data (Fig 1D). We conclude that, while a type II neural medium could produce sustained traveling waves with a large difference in the timescales of excitation and inhibition, such differences were biologically unreasonable and the resulting medium could not support graded oscillations.

### Wave propagation in a type I spatial medium

**Fig 4:**
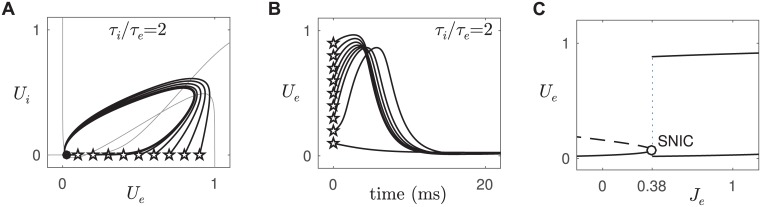
Type I excitability in an isolated pair of excitatory-inhibitory populations. All parameters were the same as for type II excitability except that the threshold of inhibition was increased from *b*_*i*_ = 4 to *b*_*i*_ = 8. *A:* Return trajectories in the phase plane. *B:* Time plots of the return trajectories. *C:* Bifurcation diagram of *U*_*e*_ versus *J*_*e*_. The critical point of the SNIC bifurcation marks the abrupt onset of large-amplitude oscillations in *U*_*e*_.

Type I excitablilty is associated with either a saddle-node bifurcation on an invariant circle (SNIC) or a subcritical Hopf bifurcation [2, 4, 12, 13]. In our model, the supercritical Hopf regime (type II excitability) is readily transformed to a SNIC (type I excitability) by shifting the inhibitory (S-shaped) nullcline rightwards until it intersects the middle branch of the excitatory (cubic) nullcline (Fig 4A). This was done by increasing the threshold of inhibition from *b*_*i*_ = 4 to *b*_*i*_ = 8. The SNIC yielded large responses to small perturbations from the rest state (Fig 4B) and its high excitability was also evident in the instantaneous onset of large oscillations in the bifurcation diagram (Fig 4C) even with modest time constants (*τ*_*i*_ = 4 ms, *τ*_*e*_ = 2 ms). The SNIC is therefore an ideal dynamical regime for sustaining propagating waves in spatial media. More importantly, the SNIC can be transformed back into the supercritical Hopf regime simply by injecting an external current into the inhibitory cell (*J*_*i*_ = 4).

We propose that optogenetic stimulation likewise transforms the excitability of cortical tissue from type I to type II. Graded gamma oscillations would thus emerge at the stimulation site via a supercritical Hopf bifurcation while the non-stimulated tissue would continue to support propagating waves via the highly excitable dynamics of the SNIC regime. We tested these predictions using the same spatial model as before but in this case we varied *J*_*e*_ while holding *J*_*i*_ = 4 fixed. This scenario represents the gradual recruitment of excitatory cells by optogenetic stimulation coinciding with the instantaneous recruitment of inhibitory cells. As always, *J*_*e*_ and *J*_*i*_ were both set to zero in the un-stimulated spatial region (|*x*|>0.25).

**Fig 5:**
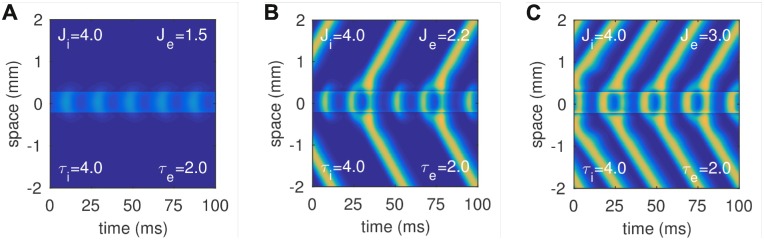
Space-time plots of LFP waves in the type I medium (*b*_*i*_ = 8). Steady stimulation was applied to both the excitatory and inhibitory cell populations near the origin (−0.25 < *x* < 0.25). The stimulation applied to the inhibitory cells (*J*_*i*_ = 4) was chosen so that the medium operated as a type II medium at the stimulation site. Absorbing boundary conditions (not shown) were imposed at |*x*| = 3 mm in all panels. *A:* Weak stimulation of the excitatory cells (*J*_*e*_ = 1.5) elicited localized gamma oscillations that emerged via a supercritical hopf bifurcation. In this case, the gamma oscillations failed to propagate as waves. *B:* Moderate stimulation (*J*_*e*_ = 2.2) evoked a propagating wave on every third gamma cycle. *C:* Strong stimulation (*J*_*e*_ = 3) evoked propagating waves on every second gamma cycle.

Low-amplitude gamma oscillations still emerged at the stimulation site, as expected, although they did not emit propagating waves when the injection current was low (Fig 5A). Waves were emitted at higher stimulation stimulation currents but not necessarily on every cycle of the gamma oscillation. For example, waves were emitted on every third gamma cycle for the case of *J*_*e*_ = 2.2 (Fig 5B) and every second gamma cycle for the case of *J*_*e*_ = 3 (Fig 5C). Importantly, the waves propagated indefinitely in the medium whenever they were emitted, as is expected of a type I excitable medium. These findings suggest that an excitable neural medium can operate in either the type I or type II regimes depending upon the influence of an external current source.

Intriguingly, one-to-one emission of waves on every gamma cycle was never observed for the model with *τ*_*i*_/*τ*_*e*_ = 2. Although it could be observed with *τ*_*i*_/*τ*_*e*_≥3.1, we considered this scenario unphysiological as it precluded the slow ramp in oscillation amplitude observed experimentally (Fig 1D), giving rise instead to a rapid rise in amplitude as shown in Fig 3E. In light of this observation, we returned to the experimental data to investigate whether the traveling ∼50 Hz gamma waves might originate from a higher harmonic oscillation localized to the stimulation site.

### Comparison to the neurophysiological data

**Fig 6:**
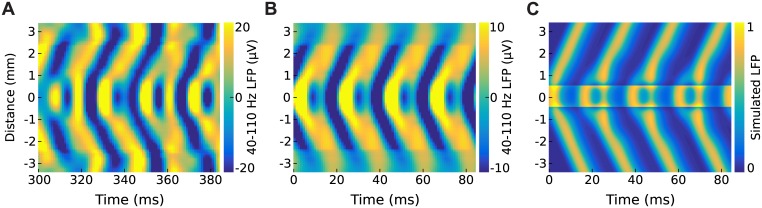
Beat skipping in the neurophysiological data versus the model. *A:* Gamma waves in single trial recordings supplied by [1]. *B:* Phase-averaged data over 60 recording trials. *C:* Simulated waves in our model where the spatial parameters (*σ*_*e*_ = 0.6 mm, *σ*_*i*_ = 0.3 mm) and temporal parameters (*τ*_*e*_ = 3.6 ms, *τ*_*i*_ = 1.8 ms) have been scaled to match the neurophysiological data.

We re-analyzed the neurophysiological data from [1] to allow direct comparison with our simulations in one spatial dimension. Non-human primate recordings and optogenetic stimulation were implemented as described in [1] under the approval of the Institutional Animal Care and Use Committee (IACUC). We observed a second peak in the LFP power spectral density at ∼100 Hz confined to electrodes within the region of direct optogenetic stimulation, suggesting that the ∼50 Hz traveling waves may indeed originate from a 2:1 resonance with local, higher-frequency gamma oscillations. For visualization, broad gamma-band (40–110 Hz) signals in the multi-electrode array were averaged according to radial distance from the site of the optogenetic source. Fig 6A shows a typical example of gamma waves being emitted from neural tissue under steady optogenetic stimulation at 6 mW. Color indicates the amplitude of the local field potential after band limiting to 40–100 Hz. Traveling gamma waves emerged within 1 mm of the optogenetic source and propagated into the surrounding tissue at 18.9 cm/s on average (SD 4.76). Remarkably, the neurophysiological data itself exhibits wave emission on every second cycle of the gamma oscillation. It is most clearly visible in the phase-averaged data (Fig 6B) where each wavefront in the ∼50 Hz oscillation is emitted on the second cycle of the ∼100 Hz oscillation at the source. In Fig 6B the LFP time-points were binned according to the phase of 48 Hz oscillations at the center of stimulation, and 45–100 Hz LFP was then averaged within each phase bin. Until now, we had only seen this behavior in simulations. Fig 6C shows the simulated results where the space constants (*σ*_*e*_ = 0.6 mm, *σ*_*i*_ = 0.3 mm) and time constants (*τ*_*e*_ = 3.6 ms, *τ*_*i*_ = 1.8 ms) of the model have been re-scaled to match the wave speed of the neurophysiological data. We find that these simulations show a remarkable agreement to the phase-averaged neurophysiological data, indicating that the 2:1 resonance is a surprising but physiologically realistic prediction of the neural field model.

### What is the relationship between *J*_*e*_ and *J*_*i*_?

It remains to ask how the inhibitory current (*J*_*i*_) might realistically vary with excitatory current (*J*_*e*_) during optogenetic stimulation. We reasoned that the optogenetically-induced currents must originate from *J*_*e*_ = 0 and *J*_*i*_ = 0 and increase smoothly with stimulation. Furthermore, the inhibitory current must saturate at *J*_*i*_ = 4 for the type I regime to be transformed to a type II regime. To this end we assumed a sigmoidal relationship,

$$J_{i}=\frac{8}{1+\exp(-\beta J_{e})}-4,$$ (7)

where *β* is an unknown slope parameter.

**Fig 7:**
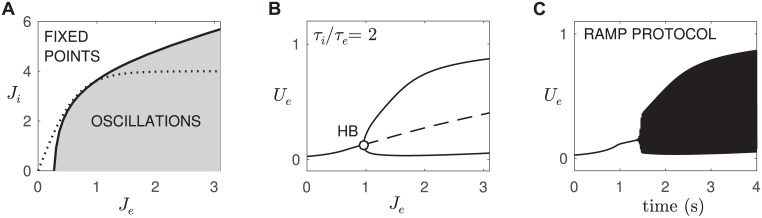
The relationship between *J*_*e*_ and *J*_*i*_ for an isolated pair of excitatory-inhibitory cells with type I excitability. *A:* The curve of Hopf bifurcation points (solid line) indicates the critical values of *J*_*e*_ and *J*_*i*_ where oscillations emerge. The dotted line represents our proposed relationship between *J*_*e*_ and *J*_*i*_ during optogenetic stimulation. It is a sigmoidal function of *J*_*e*_ that saturates at *J*_*i*_ = 4. *B:* The supercritical Hopf bifurcation under the proposed relationship between *J*_*e*_ and *J*_*i*_. Notice the slow rise in oscillation amplitude. *C:* Simulated ramp protocol under the same conditions. The excitatory current was ramped from *J*_*e*_ = 0 (*J*_*i*_ = 0) to *J*_*e*_ = 3 (*J*_*i*_ = 4) over a period of *t* = 4 seconds to mimic the ramp protocol by [1].

The curve of (*J*_*e*_, *J*_*i*_) points (Eq 7) must pass through a Hopf bifurcation where the dynamics shift from fixed points to oscillations. We used numerical continuation to map the critical values of *J*_*e*_ and *J*_*i*_ where those Hopf bifurcation points occur (Fig 7A). We chose the slope parameter *β* = 3 so that the curve of (*J*_*e*_, *J*_*i*_) points intersected the line of Hopf bifurcation points near *J*_*e*_ = 1. More pertinently, that choice allows the (*J*_*e*_, *J*_*i*_) curve to closely graze the Hopf bifurcation points in the vicinity of the intersection point. Doing so facilitates the gradual rise in the oscillation amplitude as the critical point is traversed. That slow growth in the oscillation is evident in the bifurcation diagram (Fig 7B) where *J*_*e*_ is varied while *J*_*i*_ is governed by Eq (7). It is also observed in the simulated ramp protocol (Fig 7C) where the excitatory current was slowly increased from *J*_*e*_ = 0 to *J*_*e*_ = 3 over a period of *t* = 4 seconds to mimic the ramp protocol by [1]. We argue that this slow increase in the amplitude of the gamma oscillation is what accounts for the slow rise in the power of the gamma-band oscillations reported by [1]. The slight delay in the onset of the oscillations in the simulated ramp (Fig 7C) is due to critical slowing in the vicinity of the Hopf bifurcation. We expect that the neural tissue would likewise exhibit critical slowing. This prediction could be tested by comparing the time course of optogenetically-induced gamma oscillations in the ramp-down protocol versus the existing ramp-up protocol.

## Discussion

Lu et al [1] correctly recognized that the onset of optogenetically-induced gamma oscillations in their experimental setup and protocols is consistent with a supercritical Hopf bifurcation. The supercritical Hopf is the hallmark of a type II excitable system. Yet they also observed wave propagation which is typically associated with type I excitability. We used a Wilson-Cowan [7, 8] neural field model to identify those conditions under which a type II excitable medium might support propagating waves like those observed in vivo. We found that the model could only do so if the time course of inhibition was substantially slower than that of excitation (*τ*_*i*_/*τ*_*e*_ ≳ 12). Under such conditions, the oscillation amplitude grows explosively as the stimulation is increased. However such explosive growth is inconsistent with the slow rise in the gamma power observed by [1] in their stimulus ramp protocol. We conclude that a type II excitable medium with the dynamical properties reported by [1] is not capable of supporting propagating waves.

We instead propose that the neural tissue typically operates as a type I excitable medium and that it can be locally transformed into type II by the very act of optogenetic stimulation activating the inhibitory interneurons. Type I neural medium are exemplified by arbitrarily small firing frequencies and large responses to small perturbations—characteristics which are ideal for sustaining indefinite propagating waves. Our analysis shows that a Wilson-Cowan neural field with type I excitability due to a SNIC bifurcation can be readily transformed into a type II excitable system by strongly stimulating the inhibitory cells. Simulations confirm that under these conditions the model reproduces the graded increase in gamma oscillations at the stimulation site while simultaneously supporting wave propagation in the regions beyond the stimulation site. The model thus accounts for the two major observations of [1] and resolves the apparent contradiction of type I and type II excitability.

The question remains as to how optogenetic stimulation might differentially activate inhibitory and excitatory neurons as our model suggests. Analysis of the phase plane shows that the inhibitory neurons must be recruited early and strongly in order to shift the nullcline of *U*_*i*_ from the left hand branch of the nullcline of *U*_*e*_ to the right hand branch. For simplicity, we have presented this in our model as a sigmoidal relationship (Eq 7) between the external currents *J*_*e*_ and *J*_*i*_ that represent the ionic currents induced by optogenetic stimulation. It is known that the optogenetic construct (CaMKII alpha promoter and AAv5 viral vector) used by Lu et al [1] expresses primarily in layer 5 pyramidal (excitatory) neurons and to a lesser extent in parvalbumin-positive inhibitory interneurons [14]. However it is not clear from that work whether the optogenetic construct activates inhibitory neurons earlier than excitatory neurons as our model suggests. Further studies are required to investigate the rate at which excitatory and inhibitory neurons are recruited by optogenetic stimulation.

It is reasonable to also ask whether the neural medium can be transformed into type I excitability by shifting the dynamics from the supercritical to the subcritical Hopf regime rather than to the SNIC as we suggest. Neural fields with subcritical Hopf dynamics have previously been shown to emit solitary and N-pulse traveling waves when subjected to constant stimulation [15–17]. The bistability associated with the subcritical Hopf bifurcation allows those models to support co-existing resting state and wave pulse solutions. The resting state loses stability when a constant injection current is applied to the field, leaving only the stable wave solution. When that stimulation is applied focally, it induces a local oscillation that emits wave pulses which propagate throughout the resting medium [16]. As with the present model, the wave pulses are emitted with a range of n:m mode locking regimes when the stimulation is weak [16]. In our model and theirs, the mode locking is due to the time course of the recovery variable which can block wave propagation if it remains high from a previous oscillation cycle. However the bistable models lack the supercitical Hopf dynamics needed to replicate the graded rise in the gamma oscillation observed by Lu and colleagues [1]. Moreover, it is not clear how the dynamics in those models could be transformed from subcritical to supercritical Hopf in a biologically plausible fashion. Especially since the existence of the subcritical Hopf regime seems to depend on the high gain limit of the Heaviside firing rate function, which is presumably a fixed property of the biology. Thus we regard the present model as a more compelling account of the neurophysiological data.

Interestingly, wave emission in the model of Folias and Bressloff [16] is only observed when the stimulation is ramped from high to low. In that scenario, the initial strong stimulation produces a stable stationary wave solution which is transformed into a pulsating ‘breather’ when the stimulation falls below a critical level. The pulsations become more pronounced with subsequent reductions in stimulation until the breather eventually emits traveling pulses. Yet the breather fails to materialize when the stimulation is instead ramped from low to high because of hysteresis in the bistable dynamics. Importantly, that hysteresis presents an opportunity to test the competing models against the neurophysiological data. Since our model is monostable, it predicts that waves will be emitted by optogenetic stimulation irrespective of the direction of the stimulus ramping protocol. Whereas the bistable model predicts that waves will only be emitted when the optogenetic stimulus is ramped downwards. The differences ought to distinguishable in the neurophysiological data—notwithstanding the effects of critical slowing mentioned earlier.

Unfortunately, Lu and colleagues [1] did not conduct their ramp protocol in both directions, so a new experiment must be conducted to test the hypothesis. Nonetheless, our re-analysis of their data has already confirmed some predictions of the present model. Notably, the 2:1 mode-locking of the wave emission correctly predicted the existence of ∼100 Hz oscillations at the stimulation site with the ∼50 Hz gamma oscillations being restricted to the peripheral tissue. Although we acknowledge that the same prediction also follows from the model of Folias and Bressloff [16]. The same behaviors have yet to be fully investigated in spiking neuron models. Conductance-based models of recurrently connected inhibitory neurons with type I excitability have been shown to elicit gamma-band oscillations in the macroscopic network behavior [18]. That oscillation is due to the synchronization of individual neurons which do not necessarily fire on every cycle. Moreover, it emerges from the asynchronous state through a supercritical Hopf bifurcation. Hence the dynamics of the population oscillation in the spiking neuron model are consistent with that of our neural field model. Whether those oscillations can propagate in a spatially extended spiking neural network has yet to be investigated.

In summary, the present model suggests that optogenetic stimulation can locally transform the excitability of cortical tissue from type I to type II by recruiting inhibitory interneurons prior to recruiting excitatory neurons. In doing so, it accounts for the seemingly contradictory observations of traveling waves and and supercritical Hopf bifurcation dynamics in the neurophysiological data. Further neurophysiological studies are required to determine whether optogenetic stimulation does indeed differentially recruit inhibitory and excitatory neurons as we propose. In addition, our model makes the testable prediction that gamma waves are induced at the same critical level of optical stimulation, irrespective of whether the stimulation is ramped upwards or downwards. This prediction allows our model to be empirically distinguished from the bistable model of Folias and Bressloff [16] which predicts that wave-emitting breathers only arise when the optogenetic stimulation is ramped downwards.
